# The Influence of the Gender Asterisk (“Gendersternchen”) on Comprehensibility and Interest

**DOI:** 10.3389/fpsyg.2021.760062

**Published:** 2021-12-14

**Authors:** Marcus C. G. Friedrich, Veronika Drößler, Nicole Oberlehberg, Elke Heise

**Affiliations:** Institute of Educational Psychology, Technische Universität Braunschweig, Braunschweig, Germany

**Keywords:** gender-fair language, masculine generics, grammatical gender, queer linguistics, comprehensibility, readability, interest

## Abstract

Recently, the gender asterisk (“Gendersternchen”) has become more widespread in grammatical gender languages in order to represent all genders. Such gender-fair language is intended to help better address women and other genders and make their interests and achievements more visible. Critics often argue this would make the language less comprehensible and less aesthetically appealing. Two experiments examined the effects of the gender asterisk on text comprehensibility, aesthetic perception, and interest. *N* = 159 and *N* = 127 participants were randomly provided with a text in either masculine-only form or alternatively in gender-fair language with the gender asterisk. The results of the first experiment showed no impairment of comprehensibility and aesthetic evaluation of the texts by the gender asterisk and no effect on interest in the game, while the second experiment showed significant impairments of comprehensibility, aesthetic evaluation, and interest in the game by the gender asterisk. The proportion of singular forms is discussed as a possible explanation for the different results. Experiment 1 predominantly used plural forms like die Spieler*innen (∼“the fe*male players”) and did not include forms such as der*die Spieler*in (∼“the*the fe*male player”), whereas Experiment 2 included many such more complex singular forms. We argue that this issue might be crucial, and that it deserves full attention in future studies.

## Introduction

Languages differ widely regarding the representation of gender. Gygax et al. (2019) distinguish (1) genderless languages, (2) genderless languages with a few traces of grammatical gender, (3) natural gender languages, (4) languages with a combination of grammatical gender and natural gender, and (5) grammatical gender languages. In genderless languages such as Finnish or Turkish, there are only a few nouns and pronouns that are assigned to a gender. In genderless languages with a few traces of grammatical gender like Basque or Oriya, most personal nouns and pronouns are not assigned to a specific gender; there are, however, some nouns, adjectives, and verbal forms in which the gender of the persons referred to is indicated using suffixes. In natural gender languages like English or Swedish, most pronouns are assigned to a gender, but most nouns are not. In languages with a combination of grammatical gender and natural gender like Dutch or Norwegian, some nouns refer to a specific gender, but the majority of nouns referring to humans do not refer to a specific gender, while pronouns typically are assigned to a specific gender. Finally, in grammatical gender languages such as Czech, French, German, or Spanish, nouns and most pronouns are assigned to a gender and the feminine forms are often derivations of the masculine forms. In German, for example, many feminine forms are formed by adding the suffix *-in* to the masculine form in the singular and the suffix *-innen* in the plural, e.g., *Lehrer, Lehrerin* for “teacher (masculine), teacher (female)” and *Lehrer, Lehrerinnen* for “teachers (masculine), teachers (female).”

In most grammatical gender languages, it is common practice to use masculine-only forms to refer to referents of all genders. For example, in German, *Spieler*, “player (masculine),” would be used for players of all genders. This practice is often called “masculine generics.” The term “masculine generics,” however, has been criticized. The use of masculine-only forms to refer to all or any genders or to refer to males only has the same linguistic form in either case. Whether the form is meant generically or specifically is subject to uncertainty in each case. In the following, we will therefore speak of masculine-only forms. The practice to use masculine-only forms to represent all or any genders has inherent problems: if masculine-only forms are used, readers or listeners can be sure that this masculine form refers to male referents, but they cannot be equally sure that the masculine form also refers to referents of other genders (Gabriel and Gygax, 2016). Critics of this practice therefore argue that the use of masculine-only forms makes women and other genders, their achievements and interests, less visible. These assumptions are supported by an overwhelming number of empirical studies (see sections “Studies of the Effects of Masculine-Only Forms and Gender-Fair Language on Mental Representations and Behavior” and “Influence of Gender-Fair Language on Commitment and Interest in Occupations”). Therefore, since at least the 1970s there has been a controversy about the use of masculine-only forms or gender-fair language in the designation of persons in texts (Braun et al., 2005).

In general, there are two strategies to ensure gender-fair language: neutralization and feminization (see Sczesny et al., 2016; Gabriel et al., 2018). Neutralization strategies consist in using gender-neutral forms, for example, nominalized participles in plural forms, e.g., *Spielende* for “those who play.” Feminization strategies consist in making female forms more visible by using pair-forms [e.g., *Spielerin oder Spieler*, “player (female) or player (masculine)”], so-called capital-I forms (e.g., *SpielerInnen*, “∼feMale players”), or slash forms (e.g., *Spieler/in*, “fe/male player”). These forms have been criticized for not adequately representing the genders beyond the male–female dichotomy (Diewald and Steinhauer, 2020). Recently, therefore, the gender gap (e.g., *Spieler_in*, ∼“fe_male player”), the colon (e.g., *Spieler:in*, ∼“fe:male player”), and the gender asterisk (in German “Gendersternchen,” e.g., Spieler*in, ∼“fe*male player”) and other forms have been proposed (Diewald and Steinhauer, 2020). These forms are intended to explicitly typographically include genders other than male and female (Kolek, 2019). So far, however, there has been little research on the effects of these more recent forms of gender-fair language. Studies on this issue are particularly relevant to German because in 2017, the German Civil Status Act has added the new category “diverse” to the two previous categories “male” and “female” (Bundesverfassungsgericht, 2017). As a result, politicians and the German Orthography Council are concerned with the question of how all genders should be represented in language (Rat für deutsche Rechtschreibung, 2018; Kolek, 2019). One of the central criteria for evaluating the corresponding recommendations is comprehensibility, which can be defined as the ease with which readers can perform the cognitive processes necessary to build an adequate mental representation of a subject (Friedrich and Heise, 2019).

The aim of the present study is to examine the influence of the gender asterisk as a special form of gender-fair language on comprehensibility and interest. To this end, previous studies on how gender forms in language influence mental representations, commitment, interest, and behavior are presented below. Subsequently, the concept of comprehensibility and studies of the influence of gender-fair language on comprehensibility are presented. Finally, hypotheses regarding the influence of the gender asterisk on comprehensibility and interest are derived and tested in two experiments.

### Studies of the Effects of Masculine-Only Forms and Gender-Fair Language on Mental Representations and Behavior

Numerous studies using different methods show that the use of masculine forms, even when all genders are meant to be included, leads to a mental overrepresentation of men (male bias), while the use of gender-fair language, on the other hand, leads to more balanced gender perceptions (e.g., Gabriel et al., 2008; Sato et al., 2016a,b; Esaulova et al., 2017; Misersky et al., 2019; for an overview see for example Braun et al., 2005) and helps to reduce gender stereotypical thinking (Kollmayer et al., 2018). Recently, an experiment by Lindqvist et al. (2019) showed that pair forms and newly created gender-neutral pronouns such as “hen” in Swedish or “ze” in English lead to more balanced representations of the genders than masculine forms or traditional neutral terms. The authors particularly advocate newly created gender-neutral pronouns, since they are more inclusive of women and members of other genders.

A large number of studies show that the use of masculine forms also affects behavior. The study by Prewitt-Freilino et al. (2012) shows that gender equality within different speech communities correlates with the presence of grammatical gender within the spoken language. For example, communities that speak grammatical gender languages show greater wage gaps than communities that speak natural gender or genderless languages. These differences even show up between different language communities within one country and when plausible covariates such as form of government and religious traditions are controlled for. Since the language communities also differ in terms of characteristics other than the representation of gender within the language, further empirical (primarily experimental) evidence is needed. Indeed, there are already a number of experiments that examine respective relationships and provide further insight into the mechanisms involved. If, for example, stereotypically male occupations were presented in masculine-only forms, women were considered to have lower chances of success and to be less suitable for the job than when the occupations were presented in gender-fair language (Vervecken et al., 2013, 2015; Horvath and Sczesny, 2015).

### Influence of Gender-Fair Language on Commitment and Interest in Occupations

Gabriel and Gygax (2016) derive the following conclusions from self-categorization theory: if masculine-only forms are used to refer to all genders, it is uncertain whether women and members of other genders are included or not. It can therefore be deduced from self-categorization theory that women and members of other genders feel less addressed and therefore presumably also experience less personal relatedness, less commitment, and less interest in, for example, jobs when masculine-only forms are used to represent all genders. This assumption is also backed up by the four-phase-model of interest development by Renninger and Hidi (2016). Interest is a form of intrinsic motivation (Schiefele and Schaffner, 2016). According to Renninger and Hidi (2016), the word “interest” has two differing meanings: on the one hand, interest is a psychological state in which persons deal with a certain object. On the other hand, interest is a motivational variable, more precisely, the ongoing cognitive tendency to engage with an object. In both cases, interest can refer to concrete objects, events, or even abstract ideas or topics. The four-phase model of interest development distinguishes the following four phases: (1) triggered situational interest, (2) maintained situational interest, (3) emerging individual interest, and (4) well-developed individual interest. The first two phases are summarized as less developed interest, while the latter phases are summarized as more developed interest. Developed interest is a trait and a well-known predictor for state interest. According to the model, interest develops primarily when a person can establish a personal relationship to the object and is not exposed to major difficulties in dealing with the object. If the assumption of Gabriel and Gygax (2016) that women and persons of other genders feel themselves better addressed by gender-fair language than by masculine-only forms is correct, it can therefore be assumed that texts in gender-fair language arouse more interest in, for example, jobs, especially among women and other genders than texts with masculine-only forms. These assumptions were examined by several studies presented below.

Bem and Bem (1973) conducted two classic experiments in which they examined the effect of job advertisements; the job advertisements either contained gender stereotypes, gender-related job titles (e.g., frameman) and pronouns or they were neutral with regard to gender (e.g., frameworker). The results showed that people were less interested to apply for a particular job when the advertisements were directed at a different gender than the respective individual’s. In two experiments, Vervecken and Hannover (2015) compared the influence of masculine-only and gender-fair job titles. The results show that even elementary school students considered typical male occupations such as engineering to be more difficult and reported lower self-efficacy expectations for these occupations when they were presented in masculine forms than when the same occupations were presented in gender-fair language. These effects occurred among both male and female students. No corresponding effects were found for typically female and gender-neutral occupations. Comparable effects were also shown in students when comparing two webpage designs for an introductory computer science course: while no differences were found for men, women showed more sense of belonging, interest in the course, and interest in the study of computer science in general when neutral images and aesthetic features were used than when images and aesthetic features were used that corresponded more to male interests (Metaxa-Kakavouli et al., 2018). Likewise, a recent experiment showed that women considered themselves more suitable, were more interested in a program for entrepreneurship, a typically male domain, and had higher intentions to participate in the program if the advertising for it showed gender-neutral images or women and used pair forms than if the advertising showed men and/or used only masculine forms (Hentschel et al., 2018). These findings are further supported by three studies with simulated job interviews. Female applicants showed less sense of belonging, less motivation to work in a certain job, and less expected identification with the job when only masculine forms were used in the interview than when the same interview was conducted using gender-fair language (Stout and Dasgupta, 2011).

Previous studies on the effects of gender-fair language on interest and commitment have focused primarily on interest in jobs and commitment to a company. These are highly relevant findings. It is unclear, however, whether these effects can be generalized to other contexts such as the leisure context. Choosing a job is usually a lengthy process with long-term and far-reaching consequences (Gottfredson, 1981). The present study aims to examine the influence of gender-fair language on interest in other fields. For this purpose, a leisure context, more precisely board games and sports, was used. Board and card games are a widespread hobby that promotes content knowledge, the comprehension of complex concepts, mathematical thinking, communication skills, and social interaction (Bayeck, 2020). Board and card games are a growing economic sector, which in Germany, for example, had a turnover of about 594 million euros in 2019 (Wenzel, 2020). It is essential for game publishers to generate interest in their games. To our knowledge, there are no studies on whether there are associations between board games or specific types of board games and gender. It is therefore a relevant question for the board game industry whether or not instructions in gender-fair language lead to more interest in a game overall. Sport, on the other hand, is associated with health and well-being, among other things, and different sports are associated with different genders (Eccles and Harold, 1991). Contact sports, for example, can be considered stereotypically male sports. Instructions for board games and sports, however, are usually not written in gender-fair language and may thus generate unnecessarily reduced interest in the respective game. This study therefore examines whether the results regarding the influence of gender-fair language on interest and commitment in a vocational context can be generalized to apply to interest in other fields, namely board games (Experiment 1) and contact sports (Experiment 2).

Overall, the evidence so far suggests that gender-fair language can help to reduce gender inequalities and injustices. Nevertheless, gender-fair language is subject to intense and often emotional criticism (Vergoossen et al., 2020). Kolek (2019) examined articles in German newspapers regarding the gender asterisk. A common criticism of gender-fair language is the belief that it makes texts less aesthetically appealing and less comprehensible (cf. Braun et al., 2007; Stahlberg et al., 2007; Friedrich and Heise, 2019). The present study pursues the question of whether the gender asterisk as a form of gender-fair language actually makes texts less comprehensible and to what extent it fosters interest. For this purpose, the concept of text comprehensibility and studies of the influence of gender-fair language on text comprehensibility are presented below.

### Text Comprehensibility

Text comprehensibility has been studied in psychology and educational science since at least the 1920s (Kintsch and Vipond, 1979; Nuss, 2018). The concepts and instruments for the assessment of text comprehensibility can be categorized into two groups (Ballstaedt and Mandl, 1988; Friedrich and Heise, 2019). The more widespread approach considers text comprehensibility as a characteristic of texts. According to this approach, a given text is more comprehensible, for example, the shorter and more common the words used in the text are, the shorter the sentences, the simpler the syntax of the sentences, and the closer the (successive) statements of the text are linked (Klare, 1984; DuBay, 2004; Benjamin, 2012; McNamara et al., 2014). Text comprehensibility is thus equated with text complexity. According to this view, the gender asterisk increases text complexity since it is not common (yet), and makes words and sentences longer (Braun et al., 2007; Stahlberg et al., 2007; Gabriel et al., 2018).

However, this equation of text complexity and text comprehensibility has been criticized, since prior knowledge, interest, and reading skills, among others, facilitate the comprehension of texts. Kintsch and van Dijk (1978, p. 372) therefore argue that “readability cannot be considered a property of texts alone, but one of the text-reader interaction.” According to this interactionist view of text comprehensibility, a certain text is more comprehensible to a particular reader in a particular situation, the more fluently the reader can perform the processes necessary to comprehend the text. This view is supported by everyday experience: a grammar textbook might be difficult to understand for students in their first semester, but easy to understand in their last semester. The text has not changed, but possibly the prior knowledge, interest, reading competence, and reading strategies of the readers have. In order to check whether the gender asterisk affects the comprehensibility of texts, one must examine whether the ease with which the processes of text comprehension are carried out is in fact lower for texts with gender asterisks than for texts with masculine-only forms. It is of particular interest whether the gender asterisk makes it more difficult to assign meaning to the words of the text and to decode the syntax of the sentences (cf. Braun et al., 2007; Stahlberg et al., 2007; Friedrich and Heise, 2019). So far, however, there is a lack of studies of this issue.

### Studies of the Possible Influence of Gender-Fair Language on Comprehensibility

Several experiments investigated the influence of gender-fair language on comprehensibility (for an overview see Friedrich and Heise, 2019). All these studies followed the interactionist view of comprehensibility, according to which comprehensibility depends on characteristics of both the text and the reader (see section “Text Comprehensibility”), and measured comprehensibility accordingly using questionnaires or sometimes eye movements. Overall, the studies indicate the following: comprehensibility was not impaired by pair forms [e.g., *Kundinnen und Kunden*, “customers (male) and customers (female)”; Rothmund and Christmann, 2002; Braun et al., 2007; Blake and Klimmt, 2010; Friedrich and Heise, 2019], capital-I-forms (e.g., *KundInnen*, ∼“feMale customers”; Braun et al., 2007; Blake and Klimmt, 2010), or neutral forms (e.g., Personen, “persons” or neutral forms like Projektleitung, “project management”; Rothmund and Christmann, 2002; Steiger-Loerbroks and von Stockhausen, 2014; Pöschko and Prieler, 2018) but by slash forms (e.g., *Kund/inn/en*, ∼“fe/male customers”; Klimmt et al., 2008; Pöschko and Prieler, 2018); furthermore, gender-fair language tended to be evaluated as less aesthetically pleasing (Rothmund and Christmann, 2002; Klimmt et al., 2008; Friedrich and Heise, 2019). Capital-I-forms, slash forms, and the gender asterisk are each shortened pair forms (Diewald and Steinhauer, 2020). When using capital-I-forms the female form is used and the “I” is capitalized instead of lowercase to indicate that both men and women are meant. The official German orthography allows capital letters only at the beginning of words and the capital-I also seems to promote a so-called female bias (e.g., Heise, 2000). In the case of slash forms and the gender asterisk a symbol is placed between the word stem and the feminine ending in each case. Since slash forms have been shown to impair comprehensibility critics could reasonably argue that the gender asterisk also should impair comprehensibility.

### Hypotheses

Our aim was to test the critics’ claims that the use of the gender asterisk impairs the general subjective comprehensibility of texts (H_*comprehensibility*_), the ease with which readers can ascribe meaning to the words of a text (H_*word_difficulty*_), the ease with which readers can decode the syntax of the sentences of a text (H_*sentence_difficulty*_), and the aesthetic appeal of texts (H_*aesthetic_appeal*_). Effects on other aspects of comprehensibility, such as the effort needed for reorganization or the ease of building a mental model, were not tested since there is no indication from the literature that gender-fair language impairs these variables (cf. Friedrich and Heise, 2019). Based on Gabriel and Gygax’s (2016) considerations of self-determination theory, it can be assumed that gender-fair language leads to people of all genders feeling equally addressed. As a result, the interest of women and members of other genders, and therefore interest overall, should increase. The experiments therefore investigated whether the use of the gender asterisk increases the interest in the game described in the text (H_*interest*_). Two experiments were conducted to test these hypotheses. Experiment 1 used an instruction for a board game, while Experiment 2 used an instruction for a contact sport.

## Experiment 1

### Method

#### Participants

This experiment was conducted in German and was approved by the Ethics Committee of Faculty 2 of the TU Braunschweig (identification number BA_2020-11). One hundred and fifty nine participants (119 female, 40 male, 0 diverse) volunteered for the study. Their mean age was *M* = 31.40 years (SD = 10.27, range = ∼18 to ∼55 years). Subjects were recruited via various e-mail lists for a study of how people think and feel when reading texts. Psychology students were eligible to receive credits for participating in the study. All other participants had the opportunity to take part in a lottery for gift certificates worth 20 euros each.

#### Materials

As text material, a summary of the board game manual “Citadels” was used. The text consisted of 462 words. Example sentence: *In Citadels wetteifern die Spieler darum Baumeister des Königreichs zu werden, indem sie beeindruckende mittelalterliche Städte erbauen* (“In Citadels, players compete to become the kingdom’s master builder by building impressive medieval cities”). Twenty text passages contained words like *die Spieler*, “the players (masculine).” This text will be referred to as “masculine-only forms text” (MO). This text was revised to ensure a gender-fair language by systematically replacing all masculine-only forms, e.g., *die Spieler*, “the players (masculine),” with gender asterisk-forms, e.g., die Spieler*innen, ∼“the fe*male players.” A total of 17 plural forms (15 times Spieler*innen, ∼“fe*male players” and 2 times Mitspieler*innen, ∼“other fe*male players”) and 3 singular forms (two times Baumeister*in ∼“fe*male master builder,” once Startspieler*in, ∼“fe*male start player”) were manipulated. Example sentence: In Citadels wetteifern die Spieler*innen darum Baumeister*in des Königreichs zu werden, indem sie beeindruckende mittelalterliche Städte erbauen (∼“In Citadels, fe*male players compete to become the kingdom’s fe*male master builder by building impressive medieval cities”). This text consisted of 462 words and will be referred to as “gender asterisk text” (GA). The schematic replacement of masculine-only forms by gender asterisk-forms may be considered clumsy and lacking a feel for language. It was chosen purposefully in order to provoke the maximum possible impairments by this strategy.

#### Instruments

Comprehensibility measures were assessed using the comprehensibility questionnaire from Friedrich (2017). The questionnaire was based on a reinterpretation of Kintsch and Vipond’s (1979) concept of comprehensibility on the basis of the construction-integration model of Kintsch (1998) and supplemented with the concepts of comprehensibility of Langer et al. (1974) and Gagné and Bell (1981). Friedrich (2017) could show that the questionnaire reliably assesses seven factors of comprehensibility, namely subjective comprehensibility, word difficulty, sentence difficulty, argument density, effort needed for reorganization, clarity of representation, and variety of language use (the concept underlying the questionnaire considers aesthetic appeal as a part of comprehensibility). Each scale consists of three items utilizing a 5-point Likert scale, ranging from 1 = *stimmt nicht*, “I disagree,” to 5 = *stimmt genau*, “I fully agree.” Friedrich (2017) conducted a series of experiments to test several validity hypotheses in accordance with the concept of validity from American Educational Research Association [AERA] et al. (2014). A meta-analysis of these experiments yielded positive results regarding the scales for the purpose of correlational studies and group difference testing. The questionnaire was validated for university students and pupils.

In the present experiment, only those scales from the questionnaire were used for which hypotheses had been formulated. Comprehensibility was measured using the scale *subjective comprehensibility* (3 items; example item: *Ich fand den Text verständlich*, “I thought the text was comprehensible”). The ease of ascribing meaning to words was measured using the scale *word difficulty* (3 items; sample item: *Bei manchen Wörtern war ich mir nicht sicher, was sie bedeuten*, “For some words, I was not sure what they meant”). The ease of decoding the syntactical structure of the text’s sentences was measured by the scale *sentence difficulty* (3 items; sample item: *Die Sätze waren kompliziert gebaut*, “The sentences had a complicated structure”). The text’s aesthetic appeal to the readers was measured by the scale *variety of language use* (3 items; sample item: *Ich fand die Sprache lebhaft*, “I found the language lively”).

Interest regarding the board game described in the text was measured using an adapted version of the *interest after reading* scale from Kunter et al. (2002; 4 items; sample item: *Wie wichtig ist es Ihnen, noch mehr über dieses Spiel zu erfahren?* “How important is it to you to learn more about this game?”).

Interest regarding board games in general was measured as a covariate using an adapted version of the *interest after reading* scale from Kunter et al. (2002; 4 items; example item: *Macht es Ihnen generell Spaß Brettspiele zu spielen?* “Do you generally enjoy playing board games?”). Both interest scales utilized a 5-point Likert scale, ranging from 1 = *überhaupt nicht*, “not at all,” to 5 = *sehr*, “very.”

#### Procedure

The experiment was based on a between-subjects design with the factor language form (text with masculine-only forms, MO, vs. text with gender asterisk-forms, GA). The study was conducted as an online study. After the instruction, participants read one of the two randomly assigned versions of the text. Subsequently they answered the comprehensibility questionnaire and a questionnaire recording the background variables prior knowledge, age, gender, and level of education.

#### Statistical Analysis

Hypotheses were tested using ANCOVAs with the factor language form (MO vs. GA) and interest in board games in general as a covariate. α was set to 0.05. An *a priori* power analysis revealed, that thereby small effects of *f* = 0.22 could be detected with a power of 1 − β = 0.80.

### Results

Table 1 shows the means, standard deviations, and internal consistencies for the scales as well as their intercorrelations. With internal consistencies between Cronbach’s α = 0.75 and 0.91, the scales proved to be acceptable to excellent.

**TABLE 1:** Psychometric properties and intercorrelations between the variables in Experiment 1.

Measure (number of items)	2	3	4	5	6	*M* (SD)	*N*	α
1. Subjective comprehensibility (3)^1^	−0.39*	−0.72*	0.59*	0.50*	0.18*	3.44 (1.02)	159	0.87
2. Word difficulty (3)^1^		0.27*	−0.14*	−0.17*	−0.23*	1.59 (0.67)	158	0.75
3. Sentence difficulty (3)^1^			−0.45*	−0.45*	−0.02	2.45 (0.95)	159	0.87
4. Variety of language use (3)^1^				0.58*	0.19*	2.78 (0.90)	159	0.81
5. Interest in the game (4)^2^					0.44*	2.74 (0.92)	159	0.87
6. General interest in board games (3)^2^						3.85 (0.92)	159	0.91

*M, mean; SD, standard deviation. Scale range: ^1^1 = stimmt nicht, “I disagree”; 5 = stimmt genau, “I fully agree.” ^2^1 = überhaupt nicht, “not at all”; 5 = sehr, “very.”*

*p < 0.05.

#### Inferential Statistics

First, it was examined whether the groups differed with regard to the control variables. The groups did not differ significantly with respect to age (Somers *d*_*YX*_ = 0.02, χ^2^ = 3.19, *df* = 5, *p* = 0.67), gender (Φ = 0.11, χ^2^ = 1.76, *df* = 1, *p* = 0.18), level of education (Somers *d*_*YX*_ = 0.10, χ^2^ = 6.86, *df* = 8, *p* = 0.55), prior knowledge (Somers *d*_*YX*_ = 0.07, χ^2^ = 4.50, *df* = 4, *p* = 0.34), or interest in board games in general (*d* = 0.26, *t*_*emp*_ = 1.63, *df* = 157, *p* = 0.11). The following results each report the influence of language form (MO vs. GA) on the dependent variable under control of interest in board games in general. Table 2 shows the means and standard deviations for both texts.

**TABLE 2:** Descriptive statistics for the two texts in Experiment 1.

	Masculine-only forms (*n* = 77)	Gender asterisk (*n* = 82)

Scale	*M* (SD)	*M* (SD)
Subjective comprehensibility^1^	3.25 (1.09)	3.63 (0.91)
Word difficulty^1^	1.66 (0.76)	1.53 (0.57)
Sentence difficulty^1^	2.64 (1.01)	2.27 (0.86)
Variety of language use^1^	2.58 (0.94)	2.96 (0.82)
Interest in the game^2^	2.59 (0.96)	2.88 (0.87)
General interest in board games^2^	3.73 (0.99)	3.97 (0.84)

*M, mean; SD, standard deviation. ^1^1 = stimmt nicht, “I disagree”; 5 = stimmt genau, “I agree.” ^2^1 = überhaupt nicht, “not at all”; 5 = sehr, “very.”*

After adjusting for interest in board games in general, there was a statistically significant effect on subjective comprehensibility, *F*(1,156) = 4.63, *p* = 0.03, partial η^2^ = 0.03. Contrary to expectations, adjusted mean squares suggest that the use of the gender asterisk fostered comprehensibility. Hypothesis H_*comprehensibility*_ is therefore rejected.

There was no statistically significant effect on word difficulty, *F*(1,156) = 0.68, *p* = 0.41, partial η^2^ < 0.01. Hypothesis H_*word_difficulty*_ is therefore rejected.

There was a statistically significant effect on sentence difficulty, *F*(1,155) = 5.94, *p* = 0.02, partial η^2^ = 0.04. In this case, however, no homogeneity of variances could be assumed. Yet – contrary to expectations – both the mean values and the adjusted means suggest, that the use of the gender asterisk decreased sentence difficulty. Hypothesis H_*sentence_difficulty*_ is thus rejected.

There was a statistically significant effect on variety of language use, *F*(1,156) = 5.83, *p* = 0.02, partial η^2^ = 0.04. Contrary to expectations, adjusted mean squares suggest that the use of the gender asterisk increased the variety of language use. Hypothesis H_*aesthetic_appeal*_ is therefore rejected.

There was no statistically significant effect on interest in the game, *F*(1,156) = 1.48, *p* = 0.23, partial η^2^ = 0.01. Hypothesis H_*interest*_ is therefore rejected.

### Discussion

This experiment investigated the question whether the use of the gender asterisk makes texts less comprehensible and leads to more interest in the subject described. Contrary to the assumptions of the critics of gender-fair language, the use of the gender asterisk tended to have a rather positive effect on subjective comprehensibility, word difficulty, and aesthetic appeal, and did not impair sentence difficulty. From self-determination theory, the hypothesis was derived that gender-fair language appeals to members of all genders and therefore promotes interest overall. Contrary to this assumption the gender asterisk did not increase interest in the game. The findings on interest in the game are inconsistent with the results regarding the influence of gender-fair language on interest and commitment in a professional context (Bem and Bem, 1973; Stout and Dasgupta, 2011; Hentschel et al., 2018; Metaxa-Kakavouli et al., 2018). Since career choice is a much longer process with significantly larger consequences, it was surprising that the effects of gender-fair language on interest from the vocational context could not be applied to board games. It is possible that the effects are smaller than could be detected with the power of the present experiment. Yet it is also possible that the effect depends on features of the context. Board game instructions are usually much more concrete than job advertisements. Therefore, it is conceivable that readers had a clear picture of what to do in the game and for whom the game was intended, and that this information needed little supplementation by gender specific schemata. In addition, board games are usually played in a familiar environment, for example, with friends, a game group, or at a convention. The players may therefore already know more about the values and the culture in which the game will take place. In addition, a game can also be quit more easily than a job, if necessary. In a professional context, however, gender-fair language may be an important indicator of the values and culture of the otherwise probably less known work environment. Finally, the effects in relation to job titles have been shown mainly in relation to stereotypically male occupations, and we are not aware of any studies on the extent to which board games or different genres of board games are typical or atypical for a specific gender. In a second experiment, therefore, the hypothesis is tested with a text on a topic with typical male connotations, namely contact sports.

The results regarding the influence of the gender asterisk on text comprehensibility, however, are overall surprising. The gender asterisk, like pair forms, neutral forms or capital-I forms, did not show a negative effect on comprehensibility. However, since both the gender asterisk and the slash forms are constructed by placing a typographical sign between the word stem or the masculine inflectional ending and a feminine inflectional ending, it was reasonable to assume that both forms of gender-fair language would have similar effects. Contrary to expectations, the use of the gender asterisk did not impair comprehensibility but, on the contrary, led to higher values regarding the comprehensibility. This raises the question of why the gender asterisk led to higher comprehensibility values. The studies in which the slash forms were tested were conducted in 2008 and 2018. It is conceivable that the various forms of gender-fair language have since then become more common and, as a result, more comprehensible. Slash forms and gender asterisks also have different meanings. Slash forms are shortened pair forms and refer only to men and women explicitly. The gender asterisk, on the other hand, explicitly includes all gender identities, including genders outside the dichotomy of men and women typographically (Diewald and Steinhauer, 2020). Yet, it is also possible that the typographic symbol of the asterisk evokes different associations than the slash. The slash might be more strongly associated with bureaucracy and perhaps separation or distinction, while the asterisk might be more associated with additional information and thus appears more inclusive of women and members of other genders. The gender asterisk was also introduced later than the slash forms. Differences in the (temporal) context of the introduction might have led to the two being perceived differently; the gender asterisk, for example, was already adopted by a major German party in 2015 (Diewald and Steinhauer, 2020). Therefore, studies of readers’ associations with the different forms of gender-fair language are desirable.

Since the gender asterisk makes the words and sentences longer and thus increases text complexity, it is nevertheless unclear why it has had a positive effect on comprehensibility contrary to expectations. The hypotheses are therefore tested again in Experiment 2. In order to test the generalizability of the results, Experiment 2 makes the manipulated passages more diverse in terms of the manipulated words, the numerus of the words, and manipulated dependent words such as articles and adjectives. Since the effects of gender-fair language on interest in occupations have been shown to be most pronounced for typically male occupations, Experiment 2 also used a text on a stereotypically male topic, namely contact sports.

## Experiment 2

### Method

#### Participants

This experiment was also conducted in German and was approved by the Ethics Committee of Faculty 2 of the TU Braunschweig (identification number BA_2021-03). One hundred and twenty-seven participants (85 female, 41 male, 1 diverse) volunteered for the study. Their mean age was *M* = 26.93 years (SD = 11.79, range = 18–69 years). Subjects were recruited *via* social media for a study on comprehensibility. Psychology students were eligible to receive credits for participating in the study.

#### Materials

A summary of the rules for the contact sport Kabaddi, which is little known in Germany, was used as text material. The text consisted of 471 words. Example sentence: *Jede Mannschaft schickt abwechselnd einen Angreifer – den sogenannten Raider – in die gegnerische Mannschaft* (“Each team takes turns sending an attacker – called the raider – to the opposing team”). Forty-eight text passages contained words like *der Spieler*, “the player (masculine).” This text will be referred to as “masculine-only text” (MO). This text was revised to ensure a gender-fair language by systematically replacing all masculine-only forms, e.g., *der Spieler*, “the player (masculine),” with gender asterisk-forms, e.g., der*die Spieler*in, ∼“the*the fe*male player.” A total of 17 plural forms (e.g., die Gegner*innen, ∼“the fe*male opponents”) and 31 singular forms were manipulated. The manipulated singular forms were in 20 cases nouns (e.g., der*die Angreifer*in, ∼“the*the fe*male attacker”) and in 11 cases pronouns (e.g., er*sie, “he*she,” or seine*ihre, “his*her”). Example sentence: Jede Mannschaft schickt abwechselnd eine*n Angreifer*in – die*den sogenannte*n Raider*in – in die gegnerische Mannschaft (∼“Each team takes turns sending a*a fe*male attacker – called the*the fe*male raider – to the opposing team”). This text consisted of 471 words and will be referred to as “gender asterisk text” (GA).

#### Instruments

Comprehensibility measures were again assessed using the comprehensibility questionnaire from Friedrich (2017, cf. Experiment 1). Comprehensibility was measured using the scale *subjective comprehensibility* (3 items). The ease of ascribing meaning to words was measured using the scale *word difficulty* (3 items). The ease of decoding the syntactical structure of the text’s sentences was measured by the scale *sentence difficulty* (3 items). The text’s aesthetic appeal to the readers was measured by the scale *variety of language use* (3 items).

Interest regarding the contact sport described in the text was again measured using an adapted version of the *interest after reading* scale from Kunter et al. (2002; 4 items; cf. Experiment 1).

Interest regarding contact sports in general was measured as a covariate using an adapted version of the *interest after reading* scale from Kunter et al. (2002; 4 items; cf. Experiment 1).

#### Procedure

The experiment was based on a between-subjects design with the factor language form (text with masculine-only forms, MO, vs. text with gender asterisk-forms, GA). The study was conducted as an online study. After the instruction, participants answered the scale on interest regarding contact sports in general before reading one of the two randomly assigned versions of the text. Subsequently they answered the comprehensibility questionnaire and a questionnaire recording the background variables prior knowledge, age, gender, and level of education.

#### Statistical Analysis

Hypotheses were again tested using ANCOVAs with the factor language form (MO vs. GA) and interest in contact sports as a covariate. α was again set to 0.05. An *a priori* power analysis revealed, that thereby medium effects of *f* = 0.25 could be detected with a power of 1 − β = 0.80.

### Results

Table 3 shows the means, standard deviations, and internal consistencies for the scales as well as their intercorrelations. With internal consistencies between Cronbach’s α = 0.81 and 0.92, the scales proved to be good or excellent.

**TABLE 3:** Psychometric properties and intercorrelations between the variables in Experiment 2.

Measure (number of items)	2	3	4	5	6	*M* (SD)	*N*	α
1. Subjective comprehensibility (3)^1^	−0.55*	−0.81*	0.33*	0.41*	0.07	3.73 (0.96)	127	0.88
2. Word difficulty (3)^1^		0.41*	−0.09	−0.29*	−0.15*	2.20 (0.96)	124	0.84
3. Sentence difficulty (3)^1^			−0.28*	−0.37*	−0.10	2.39 (0.87)	126	0.81
4. Variety of language use (3)^1^				0.54*	0.20*	3.11 (0.89)	127	0.81
5. Interest in the game (4)^2^					0.52*	2.40 (0.89)	127	0.92
6. General interest in contact sports (3)^2^						2.40 (0.90)	127	0.88

*M, mean; SD, standard deviation. Scale range: ^1^1 = stimmt nicht, “I disagree”; 5 = stimmt genau, “I fully agree.” ^2^1 = überhaupt nicht, “not at all”; 5 = sehr, “very.”*

*p < 0.05.

#### Inferential Statistics

First, it was examined whether the groups differed with regard to the control variables. The two groups did not differ significantly with respect to age (*d* = 0.14, *t*_*emp*_ = 0.77, *df* = 125, *p* = 0.44), gender (*Cramer’s V* = 0.11, χ^2^ = 1.60, *df* = 2, *p* = 0.45), level of education (Somers *d*_*YX*_ = 0.14, χ^2^ = 3.94, *df* = 3, *p* = 0.27), prior knowledge (Somers *d*_*YX*_ = 0.10, χ^2^ = 3.01, *df* = 2, *p* = 0.22), or interest in contact sports in general (*d* = 0.02, *t*_*emp*_ = 0.10, *df* = 125, *p* = 0.92). Even though, the hypotheses were tested using ANCOVAs with the interest in contact sports in general as a covariate. Therefore, the following results each report the influence of language form (MO vs. GA) on the dependent variable under control of interest in contact sports in general. Table 4 shows the means and standard deviations for both texts.

**TABLE 4:** Descriptive statistics for the two texts in Experiment 2.

	Masculine-only forms (*n* = 66)	Gender asterisk (*n* = 61)

Scale	*M* (SD)	*M* (SD)
Subjective comprehensibility^1^	3.98 (0.76)	3.46 (1.07)
Word difficulty^1^	2.19 (0.94)	2.21 (0.99)
Sentence difficulty^1^	2.16 (0.71)	2.64 (0.96)
Variety of language use^1^	3.26 (0.92)	2.95 (0.84)
Interest in the game^1^	2.50 (0.88)	2.28 (0.90)
Interest in contact sports^2^	2.39 (0.91)	2.41 (0.89)

*M, mean; SD, standard deviation; ^1^1 = stimmt nicht, “I disagree”; 5 = stimmt genau, “I agree.” ^2^1 = überhaupt nicht, “not at all”; 5 = sehr, “very.”*

After adjusting for interest in contact sports in general, there was a statistically significant effect on subjective comprehensibility, *F*(1,124) = 10.11, *p* = 0.002, partial η^2^ = 0.08. Adjusted mean squares suggest that the use of the gender asterisk decreased comprehensibility. Hypothesis H_*comprehensibility*_ is therefore supported.

There was no statistically significant effect on word difficulty, *F*(1,121) = 0.00, *p* = 0.95, partial η^2^ = 0.00. Hypothesis H_*word_difficulty*_ is therefore rejected.

There was a statistically significant effect on sentence difficulty, *F*(1,123) = 10.48, *p* = 0.002, partial η^2^ = 0.08. Adjusted means suggest that the use of the gender asterisk increased sentence difficulty. Hypothesis H_*sentence_difficulty*_ is thus supported.

There was a statistically significant effect on variety of language use, *F*(1,124) = 4.03, *p* = 0.047, partial η^2^ = 0.03. Adjusted mean squares suggest that the use of the gender asterisk decreased the variety of language use. Hypothesis H_*aesthetic_appeal*_ is therefore supported.

There was a statistically significant effect on interest in the game, *F*(1,124) = 2.82, *p* = 0.10, partial η^2^ = 0.02. Adjusted means suggest that the use of the gender asterisk decreased interest in the contact sport described. Hypothesis H_*interest*_ is therefore rejected.

### Discussion

This experiment again investigated the question whether the use of the gender asterisk makes texts less comprehensible and leads to more interest in the subject described. While Experiment 1 showed no impairments due to the use of the gender asterisk, Experiment 2 showed clear impairments of comprehensibility and interest when the gender-asterisk was used. The different results of the two experiments regarding the comprehensibility measures and differences between the two experiments are discussed in the general discussion (see section “General Discussion”).

The results on the influence of gender-fair language on interest from the occupational context could not be replicated in Experiment 2 either. This could again be due to the fact that people usually engage in sports in a familiar environment and that they thereby rarely make long-term commitments. The negative effect of the gender asterisk on interest in the game in this experiment is also in line with fluency theory, which states that objects are evaluated more positively, the easier they can be processed by an individual (Reber and Greifeneder, 2017). Comprehensibility can be considered as a form of fluency (Graf et al., 2017). The theory thus suggests that incomprehensible texts lead to more negative evaluations of the texts themselves and the objects they describe. This is in line with the lower values regarding aesthetic appeal. Yet, these relations should be examined in more detail in future studies.

## General Discussion

To our knowledge, the present experiments are the first to investigate the question whether the use of the gender asterisk makes texts less comprehensible, but leads to more interest in the subject described. Thereby Experiment 1 showed rather positive effects of the gender asterisk on comprehensibility and aesthetic appeal and no effect on interest in the described game, while Experiment 2 showed small to medium negative effects of the gender asterisk on comprehensibility, aesthetic appeal, and interest. These different results of the two experiments are puzzling at first.

The experiments differed with regard to several variables, namely the texts, the subjects of the texts, the number of manipulated passages, and the number of plural and singular forms. Therefore, we would argue that it is not possible to clearly identify the cause of the different results. The possibility that the differences are due to the number of plural and singular forms seems particularly interesting for future studies. In many languages the plural and singular forms go hand in hand with the article used and other dependent parts of speech such as adjectives. While in English, for example, there is only the definite article “the,” in German, for example, there are three definite articles (“der,” “die,” and “das”). Which article is used depends on the grammatical gender the respective noun is assigned to, typically “die” for women in singular, “der” for men in singular, and “die” in plural forms for all genders. Thus, the plural form can easily be formed with “die” (e.g., die Spieler*innen, ∼“the fe*male players”). When using the singular, however, it is unclear which article should be used. For this reason, a text may mention both articles and connect them with an asterisk. This leads, for example, to the more complex form der*die Spieler*in, ∼“the*the fe*male player.” The text in Experiment 1 contained 3 singular forms and 17 plural forms, while the text in Experiment 2 contained 31 singular forms and 17 plural forms. While constructions like “der*die” do not occur in the text with gender asterisk in the first experiment, the text with gender asterisk in the second experiment contains 13 passages with constructions like der*die Spieler*in, ∼“the*the fe*male player,” or des*der Raider*in, ∼“the*the fe*male raider,” 11 passages with constructions like er*sie, “he*she” or seine*ihre, “his*hers,” and 4 passages with constructions like ein*e Spieler*in, ∼“a*a fe*male player.” These constructions are very uncommon in German and the differences here do not only involve a single word, but an entire phrase.

The descriptions of former studies on the comprehensibility of gender-fair language rarely clearly indicate the extent to which plural forms or singular forms were examined or manipulated. Overall, however, it seems that most studies mainly investigated plural forms (Rothmund and Christmann, 2002; Braun et al., 2007; Klimmt et al., 2008; Blake and Klimmt, 2010; Steiger-Loerbroks and von Stockhausen, 2014; Pöschko and Prieler, 2018). It seems that only Friedrich and Heise (2019) used mainly singular forms in their texts, also manipulating the articles. Future studies on the comprehensibility of gender-fair language should therefore increasingly investigate the comprehensibility of singular forms and forms such as der*die Spieler*in, ∼“the*the fe*male players.” For a comparison of the influence of plural and singular forms, future studies should use either single-factor designs with three factor levels (masculine-only forms, gender asterisk in plural forms, and gender asterisk in singular forms), or two-factor designs with the factors gender-fair language (masculine-only forms vs. gender asterisk) and grammatical number (singular vs. plural).

The present experiments are subject to a number of limitations, though. It is conceivable that the participants of the gender asterisk conditions anticipated that the study was concerned with the comprehensibility of the gender asterisk and that they at least to some degree consciously evaluated the texts more positively due to their world view or sense of social desirability. However, this possibility is countered by the fact that the study used double-blind experiments with between-subjects designs. Several freely offered comments at the end of the studies criticizing the use of the gender asterisk also indicate that at least most of these participants did not realize the purpose of the experiment. Furthermore, since the samples consisted mostly of students of psychology, humanities, and education, it is reasonable to assume that the samples studied are more familiar with the gender asterisk than other groups and that they have a rather positive attitude toward gender-fair language. Moreover, the experiments did not assess reading times. It is possible that participants who read the text in gender-fair language needed more time to reach the same level of comprehension as participants who read the text with masculine-only forms. Therefore, further studies with non-student samples and non-reactive measurement methods such as eye movements and reading times are desirable. They could, for example, test whether reading words and sentences with gender asterisks takes longer than reading words and sentences with masculine-only forms. The results of the experiments should therefore be replicated with other populations, other texts, further text types, and further measures.

Future studies should in particular focus primarily on possible differences regarding gender-fair language in plural forms and singular forms. Research should also examine whether the gender asterisk leads to balanced mental images of the genders and whether it makes discriminatory behavior less likely. Since the gender asterisk is similar to other forms of gender-fair language such as the capital-I forms (e.g., *SpielerInnen*, “∼feMale player”) and slash forms (e.g., *Spieler/in*, “fe/male player”), such effects can be expected. Since the gender asterisk is also supposed to represent genders outside the male–female dichotomy, it would be particularly interesting to test whether the gender asterisk actually has this effect regarding other genders on mental representations and behavior.

If these results are confirmed in further studies, they will support the use of the gender asterisk in plural forms. Overall, gender-fair language does not seem to affect comprehensibility as long as it is similar to the forms people are used to.

## Data Availability Statement

The raw data supporting the conclusions of this article will be made available by the authors, without undue reservation.

## Ethics Statement

The studies involving human participants were reviewed and approved by the Ethikkommission der Fakultät 2 der TU Braunschweig. The patients/participants provided their written informed consent to participate in this study.

## Author Contributions

MF prepared the data sets for the analyses, conducted the analyses, and wrote the first draft of the manuscript. VD conducted the Experiment 1. NO conducted the Experiment 2. EH scientifically supervised each step of the study, contributed to the development and the design of the experiments, and was substantially involved in the final version of the manuscript. All authors contributed to the article and approved the submitted version.

## Conflict of Interest

The authors declare that the research was conducted in the absence of any commercial or financial relationships that could be construed as a potential conflict of interest.

## Publisher’s Note

All claims expressed in this article are solely those of the authors and do not necessarily represent those of their affiliated organizations, or those of the publisher, the editors and the reviewers. Any product that may be evaluated in this article, or claim that may be made by its manufacturer, is not guaranteed or endorsed by the publisher.
